# An innovative screening method for heat stress tolerance in chickpea (*Cicer arietinum* L.)

**DOI:** 10.1016/j.mex.2026.104002

**Published:** 2026-06-11

**Authors:** S. Gurumurthy, C. Laxuman, K.K. Hazra, K.R. Soren, P.V.V. Prasad

**Affiliations:** a ICAR-National Institute of Abiotic Stress Management, Pune, Maharashtra 413115, India; b ICAR-Indian Agricultural Research Institute Regional Station, Indore, Madhya Pradesh 452001, India; c Zonal Agricultural Research Station, Kalaburagi, Karnataka 585101, India; d ICAR-Indian Institute of Pulses Research, Kanpur, Uttar Pradesh 208024, India; e ICAR-Indian Institute of Agricultural Biotechnology, Ranchi, Jharkhand 834010, India; f Department of Agronomy, Kansas State University, Manhattan 66506, Kansas, USA

**Keywords:** Deflowering, Reproductive thermotolerance, Phenotyping, Pod set, Pollen viability

## Abstract

Reliable field phenotyping for terminal heat stress (THS) tolerance in chickpea is constrained by conventional late-sowing approaches that confound reproductive stress with reduced vegetative growth. We developed and validated a deflowering (DF)-based field screening method that selectively imposes heat stress during the reproductive phase while maintaining normal vegetative vigour. Early flowers were removed to synchronize flowering and delay reproduction by 10–15 days, exposing flowering and pod set to high temperatures (>33 °C). Across two seasons and contrasting genotypes, DF maintained vegetative growth but significantly reduced pollen viability, pod set, and yield, with tolerant genotypes showing markedly lower yield penalties than susceptible ones. The method effectively discriminated reproductive thermotolerance and provides a simple, low-cost, and biologically grounded phenotyping tool for chickpea breeding under warming climates.

• A DF-based field method selectively imposes reproductive-stage heat stress without compromising vegetative growth.
• The approach reliably distinguishes heat-tolerant and susceptible chickpea genotypes under natural field conditions.

## Graphical abstract

**Figure:**
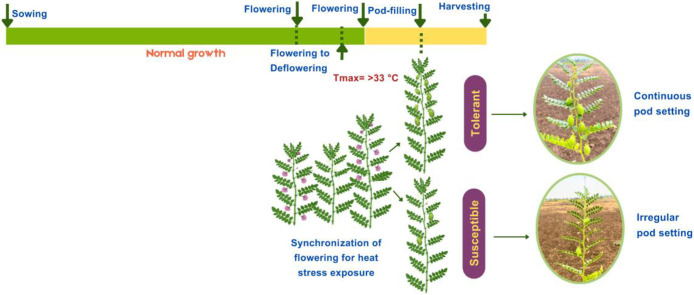

## Specifications table

**Table:** 

**Subject area**	Agricultural and Biological Sciences
**More specific subject area**	High temperature stress
**Name of your method**	Screening method for heat stress tolerance
**Name and reference of original method**	None
**Resource availability**	Data will be made available on request

## Background

Chickpea (*Cicer arietinum* L.) is the most important pulse crop in India and the second most widely grown grain legume globally, contributing significantly to food and nutritional security in semi-arid regions [1]. Rising temperatures associated with climate change pose a major threat to chickpea productivity, particularly during the reproductive phase. Elevated temperatures impair pollen viability and germination, stigma receptivity, flower retention, and pod set in chickpea and other legumes [2,3]. Among these processes, pod set is more critical than flowering in determining yield under heat stress [4]. Such disruptions ultimately reduce grain filling and yield, highlighting the urgent need to identify heat-tolerant cultivars [5].

Despite progress in understanding the physiological and genetic basis of thermotolerance [6], the development of robust heat-tolerant cultivars has been constrained by the lack of reliable field-based phenotyping methods. The commonly used delayed sowing approach exposes plants to terminal heat stress (THS) but simultaneously reduces vegetative growth, biomass, and source strength, confounding reproductive tolerance with growth limitation [7]. In addition, delayed sowing compresses crop phenology and leads to asynchronous heat exposure among genotypes of differing maturity, enabling early lines to escape stress while late lines suffer disproportionate yield losses [8,9]. Year-to-year variability in the onset and intensity of high temperatures further undermines the reliability of this approach. Although controlled-environment screening offers greater precision, it is costly, often associated with higher disease incidence, and frequently fails to capture the complexity of natural field conditions [10].

To overcome these limitations, we propose a deflowering (DF)-based field screening method as a reliable alternative for assessing reproductive heat tolerance. In this approach, early flowers are manually removed to delay flowering by 10–15 days, extending the vegetative phase and synchronizing reproductive development during periods when maximum temperatures typically exceed 33 °C. Genotypes are then evaluated based on pod set under heat stress. By preserving vegetative vigour while selectively exposing reproductive tissues to natural heat stress, the DF method enables a more accurate and consistent identification of heat-tolerant chickpea genotypes.

## Method details

### Experimental site and planting material

Field experiments were conducted during the 2023–24 and 2024–25 seasons at ICAR–National Institute of Abiotic Stress Management (NIASM), Pune, India (18.5°N, 73.8°E; 548 m above msl), characterized by a semi-arid tropical climate. Eight chickpea genotypes were evaluated, comprising four heat-tolerant (ICE 15,654 A, JG 14, IPC-06–11, MNK-1) and four heat-susceptible (Pusa-1003, Vijay, JG-63, JG 16) lines identified through prior multi-environment screening [11]. The general characteristics of the selected genotypes are mentioned (Table S1). The days to flowering of contrasting genotypes studied under normal condition are presented (Table S2).

### Experimental design, treatments and crop management

The experiment followed a factorial randomized block design (FRBD) with three replications. Treatments included non-deflowering (NS; control) and deflowering (DF), with genotypes (heat susceptible and heat tolerant) as the second factor. Each plot consisted of three rows (4 m length) with 30 cm row spacing and 10 cm plant spacing. Sowing was done in the last week of November in both years under drip irrigation. Basal fertilizer was applied at 20–50–50 kg ha⁻¹ (N–P₂O₅–K₂O).

### Deflowering protocol and heat stress imposition

**Fig. 1:**
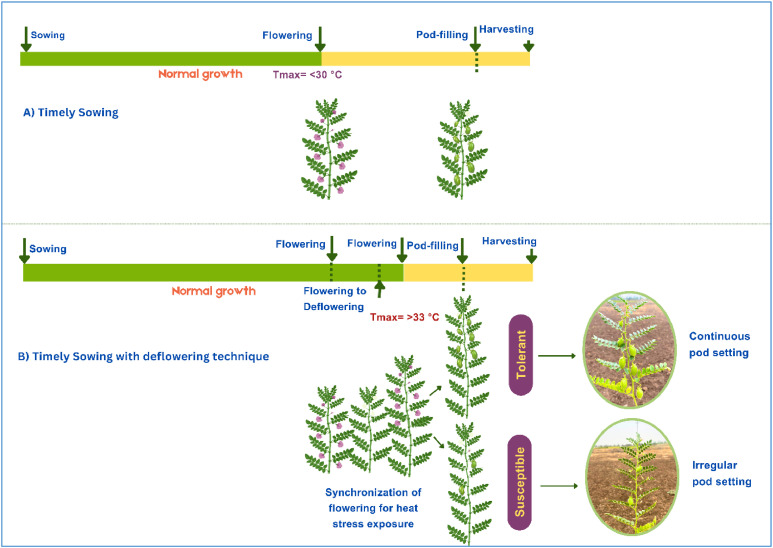
Deflowering-based technique for screening heat stress tolerance in chickpea. Under timely sowing, reproductive stages occur below 30 °C, preventing exposure to heat stress (A). In contrast, the deflowering technique synchronizes flowering under high temperatures >33 °C while maintaining normal vegetative growth, enabling precise discrimination of tolerant and susceptible genotypes based on reproductive resilience.

**Fig. 2:**
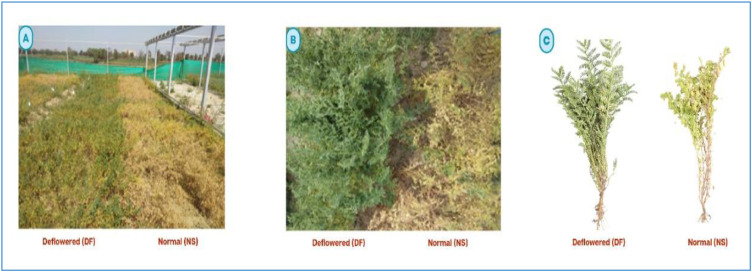
Effect of deflowering to arrest the podding and delay the onset of senescence process.

In the DF treatment, all flowers including buds, were manually removed from the onset of flowering until ambient maximum temperatures consistently exceeded 33 °C, which typically occurred by the third week of February. The standardized deflowering was carried out in three sequential phases—at 3, 7, and 10 days after the appearance of the first visible flower in each genotype—to effectively suppress early pod initiation. All plants of the deflowering treatment were carefully removed. This targeted removal of all flowers temporarily arrested reproductive development, prolonged the vegetative phase, and preserved normal vegetative vigour and source capacity. The plants were allowed to flower and set pods naturally under prevailing high-temperature conditions. Consequently, subsequent flowering and pod set were synchronized to occur late under supra-optimal temperature conditions (>33 °C), thereby ensuring uniform exposure of reproductive processes to heat stress under field conditions. Genotypes exhibiting continuous and stable pod set under heat stress were classified as heat tolerant, whereas those showing irregular pod formation or complete failure to set pods were considered heat susceptible Fig. 1. DF effectively delayed podding and the onset of reproductive senescence (Fig. 2). Screening was conducted under normal agronomic management practices without imposing additional growth constraints. To minimize transient water or mechanical stress associated with deflowering, plots were immediately irrigated after each deflowering event using a drip irrigation system at a rate of 10 L m⁻². In contrast, control plants (non-deflowered; NS) were allowed to grow normally without flower removal. Weather conditions prevailing during the crop period are presented in Fig. S1 and S2. Temperature data recorded throughout the crop season were used to determine maximum and minimum temperatures ( °C) during the flowering period and early reproductive stage. Following the methodology of Lamichaney et al. [12], mean ambient maximum and minimum temperatures were calculated for each genotype at 50% flowering (± 3 days) and at 15 days after flowering (± 3 days), enabling precise characterization of thermal exposure during critical reproductive phases.

### Evaluation metrics and statistical analysis

Phenological traits (days to 50% flowering and maturity), vegetative traits (plant height, stem dry weight, NDVI), reproductive trait and yield attributes (pollen viability, pods per plant, seeds per plant, grain yield) were recorded for each genotype using standard protocols. NDVI was measured using a GreenSeeker® sensor, and pollen viability was estimated using 2% acetocarmine staining. Data were analysed using two-way ANOVA. Treatment means were compared using LSD (*p* ≤ 0.05), and Duncan’s multiple range test (DMRT) was applied for mean separation. Percent changes under DF were calculated relative to the control for each trait.

## Method validation

**Fig. 3:**
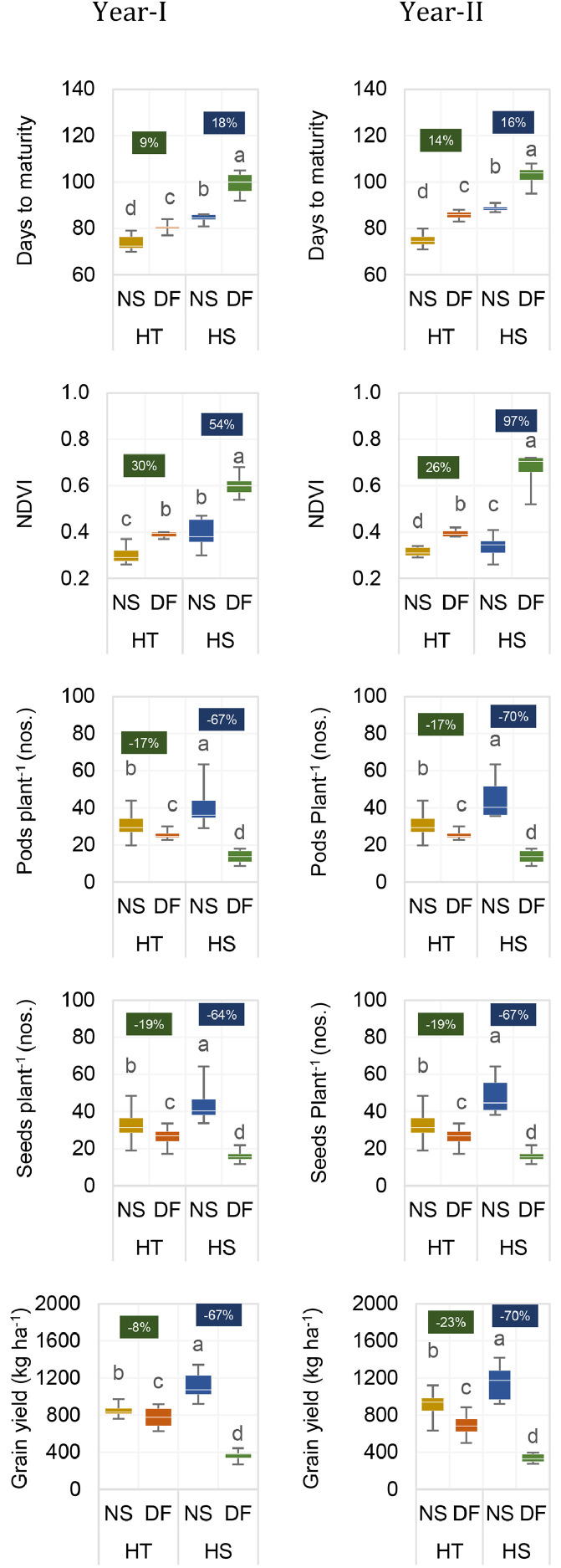
Changes (%) in plant growth and yield traits in susceptible versus tolerant groups as influenced by the deflowered (DF) over non-deflowering control (NS) in Year-I (2023–2024) and Year -II (2024–2025). NDVI Normalized Difference Vegetation Index. a-d different lowercase letters indicate significant treatment difference at p < 0.05 according to Duncan’s Multiple Range Test.

**Fig. 4:**
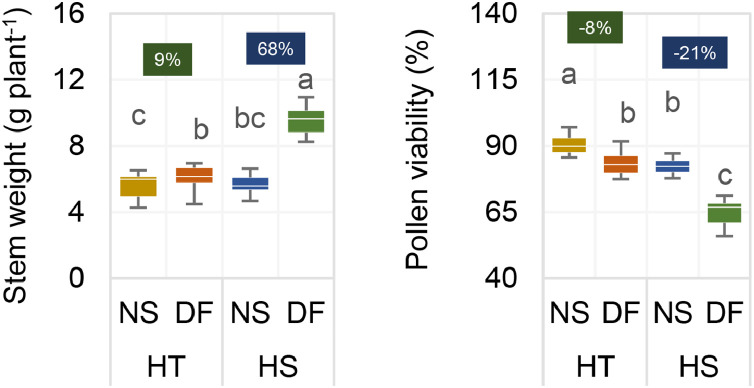
Changes (%) in stem weight and pollen viability in susceptible versus tolerant groups as influenced by the deflowered (DF) over non-deflowering control (NS) in Year -II (2024–2025). *NDVI,* Normalized Difference Vegetation Index. a-d different lowercase letter indicates significant treatment difference at p < 0.05 according to Duncan’s Multiple Range Test.

DF effectively synchronized flowering across genotypes and selectively imposed heat stress during the reproductive phase without compromising vegetative growth. Across years, DF extended crop duration by approximately 14–15%, confirming a consistent delay in reproductive onset. Vegetative growth was maintained or enhanced under DF, as evidenced by increases in plant height (∼14%), stem dry weight (∼39%), and canopy greenness (NDVI; 43–63%), indicating that vegetative development occurred under favourable thermal conditions prior to stress imposition (Table 1). In contrast, reproductive processes were markedly impaired under DF, confirming successful exposure to reproductive-stage heat stress. Pollen viability declined by an average of 12.5%, with tolerant genotypes showing only minor reductions (∼7–8%) compared with pronounced declines in susceptible genotypes (∼18–21%) (Table 2). These physiological impairments translated into significant reductions in pod number, seed number, and grain yield. Yield losses were relatively modest in heat-tolerant genotypes (8–23%) but severe in susceptible genotypes (67–70%), demonstrating the strong discriminatory capacity of the DF approach (Fig. 3, Fig. 4). The clear divergence between preserved vegetative growth and compromised reproductive performance confirms that DF specifically targets reproductive thermotolerance rather than general plant vigour or source limitation.

**Table 1:** Comparative effect of deflowering (DF) and non-deflowering treatment (NS) on NDVI, maturity duration and grain yield in different chickpea genotypes in the year 2023–24 (Year 1) and 2024–2025 (Year 2).

Genotype	NDVI				Days to maturity				Grain yield (kg ha^-1^)			
	Year 1		Year 2		Year 1		Year 2		Year 1		Year 2	
	NS	DF	NS	DF	NS	DF	NS	DF	NS	DF	NS	DF
ICE 15,654 A	0.270^i^	0.385^f^	0.335^gh^	0.380^f^	72.0^i^	80.5^fg^	74.5^h^	85.5^ef^	890^de^	887^de^	1073^bc^	626^f^
JG 14	0.275^i^	0.390^ef^	0.300^i^	0.380^f^	71.5^i^	82.0^def^	74.0^h^	85.5^ef^	790^efg^	697^fg^	940^cd^	832^de^
IPC-06–11	0.305^hi^	0.390^ef^	0.290^i^	0.400^e^	74.0h^i^	81.0^efg^	72.5^h^	87.5^de^	892^de^	876^de^	940^cd^	735^ef^
MNK-1	0.350^fgh^	0.395^ef^	0.330^h^	0.420^d^	77.5^gh^	78.5^fg^	79.0 *g*	84.5^f^	830^ef^	658 *g*	646^ef^	562^f^
Pusa-1003	0.435^de^	0.585^bc^	0.330^h^	0.710^ab^	85.0^de^	103.5^a^	90.0^d^	104.5^ab^	1136^b^	286^h^	1213^ab^	281 *g*
Vijay	0.355^fg^	0.555^c^	0.405^de^	0.700^b^	85.5^d^	97.5^b^	89.5^d^	103.0^b^	979^cd^	368^h^	960^cd^	356 *g*
JG-63	0.460^d^	0.605^b^	0.350 *g*	0.720^a^	82.0^def^	103.5^a^	87.5^de^	107.0^a^	1295^a^	389^h^	1100^bc^	344 *g*
JG 16	0.330^gh^	0.665^a^	0.270^j^	0.540^c^	85.0^de^	92.5^c^	87.5^de^	96.0^c^	1033^bc^	409^h^	1310^a^	358 *g*
Mean	0.348^B^	0.496^A^	0.326^B^	0.531^A^	79.1^B^	89.9^A^	81.8^B^	94.2^A^	981^A^	571^B^	1023^A^	512^B^
Change (%)*	+42.8		+62.8		+13.7		+15.1		−41.8		−49.9	
												
P value												
Treatment (T)	< 0.001		< 0.001		< 0.001		< 0.001		< 0.001		< 0.001	
Genotype (G)	0.003		< 0.001		0.048		< 0.001		0.002		0.004	
T × G	< 0.001		< 0.001		0.001		< 0.001		0.020		0.026	

a-i, different lowercase letters indicate significant treatment difference at *p* < 0.05 according to Duncan’s Multiple Range Test.

*Percent change in the mean value of DF treatment compared to NS.

Year-1 (2023–24), Year-2 (2024–25).

**Table 2:** Comparative effect of deflowering (DF) and non-deflowering treatment (NS) plant growth and yield attributes and pollen viability in different chickpea genotypes in the year 2024–25 (Year 2).

Genotype	Plant height (cm)		Stem weight (g)		Pollen viability (%)		Pods plant^-1^ (nos.)		Seed plant^-1^ (nos.)	
	NS	DF	NS	DF	NS	DF	NS	DF	NS	DF
ICE 15,654 A	46.3^hi^	48.8^gh^	4.38^f^	4.92^ef^	91.6^ab^	84.5^cdef^	42.3^b^	29.6^d^	46.4^b^	32.4^ef^
JG 14	43.5^j^	44.8^ij^	6.17^cd^	6.78^c^	88.3^bcd^	81.5^efg^	29.4^d^	24.9^e^	30.5^de^	26.1^h^
IPC-06–11	44.0^ij^	45.0^ij^	5.52^de^	6.01^cd^	96.6^a^	89.7^bc^	30.5^d^	24.4^e^	33.0^d^	27.6^gh^
MNK-1	52.0^de^	54.8^c^	6.30^cd^	6.58^c^	86.0^cdef^	77.8 *g*	20.7^fg^	23.3^ef^	20.5^f^	19.1^ij^
Pusa-1003	50.3^efg^	60.0^b^	4.88^ef^	10.01^a^	81.0^fg^	59.9^i^	45.3^b^	17.5 *g*	50.8^b^	20.4^ij^
Vijay	42.8^j^	49.3^fg^	5.49^de^	8.59^b^	83.5^def^	67.5^h^	61.5^a^	15.6 *g*	62.8^a^	16.2^jk^
JG-63	51.8^def^	72.0^a^	6.17^cd^	10.55^a^	78.2 *g*	69.7^h^	37.5^c^	12.1^h^	39.6^c^	14.2^k^
JG 16	46.3^hi^	54.0^cd^	6.12^cd^	8.91^b^	86.7^bcde^	61.5^i^	35.9^c^	9.4^h^	40.5^c^	13.5^k^
Mean										
Change (%)	+13.7		+38.5		−12.5		−48.3		−47.7	
										
P value										
Treatment (T)	< 0.001		< 0.001		< 0.001		< 0.001		< 0.001	
Genotype (G)	< 0.001		< 0.001		< 0.001		< 0.001		< 0.001	
T × G	< 0.001		0.022		0.010		< 0.001		< 0.001	

a-i, different lowercase letters indicate significant treatment difference at *p* < 0.05 according to Duncan’s Multiple Range Test.

*Percent change in the mean value of DF treatment compared to NS.

Year-1 (2023–24), Year-2 (2024–25).

Compared with the conventional late-sowing method, DF avoids confounding effects associated with early-season cold stress, compressed phenology, and source limitation. By maintaining vegetative growth under optimal temperatures while synchronizing flowering during periods of high temperature, DF provides a biologically grounded and field-relevant phenotyping framework for THS tolerance. The contrasting responses observed between tolerant and susceptible genotypes further validate its robustness and reproducibility. Beyond methodological robustness, DF offers clear advantages for breeding applications. The approach is simple, low-cost, and easily integrated into field-based breeding programs. Its ability to reliably reveal genotypic variation in reproductive resilience makes it suitable for multi-location testing, pre-breeding, and trait introgression. This DF-based approach may be application for screening heat tolerance in other winter legumes with indeterminate growth habit (e.g. lentil) with appropriate validation. Integration of DF-based phenotyping with molecular tools (e.g., transcriptomics and metabolomics), speed breeding, and the development of chemical or hormonal deflowering strategies could further enhance its scalability and utility for climate-resilient legume improvement.

## Limitations

Manual deflowering, although effective in manipulating reproductive timing, is highly labour-intensive and economically inefficient under field conditions. Moreover, repeated flower removal can cause mechanical injury to plants, potentially inducing unintended physiological stress and confounding treatment effects.

## Ethics statements

Not applicable.

## CRediT author statement

**S. Gurumurthy**: Conceptualization, Methodology, Writing – original draft, Visualization, Investigation. **C. Luxuman**: Writing – review & editing, **K.K. Hazra**: Data Curation, Writing – original draft, Writing – review & editing. **K.R. Soren**: Visualization, Writing – review & editing. **P.V.V. Prasad**: Writing – review & editing.

## Declaration of competing interest

The authors declare that they have no known competing financial interests or personal relationships that could have appeared to influence the work reported in this paper.

## Data Availability

Data will be made available on request.

## Appendix Supplementary materials

Supplementary material *and/or* additional information [OPTIONAL] Provided. Image, application 1
