# Supplementary material for: An innovative screening method for heat stress tolerance in chickpea (Cicer arietinum L.)
Source: MethodsX. 2026 Jun 11;17:104002. doi: 10.1016/j.mex.2026.104002 (PMC13311209; doi:10.1016/j.mex.2026.104002)
Supplement: Supplementary file 1 — Supplementary material and/or additional information [OPTIONAL] Provided. [file mmc1.docx]

**Table S1.** General characteristics of the selected genotypes used in the study

| Genotype | Type | Days to maturity | Seed type | Special characteristics | Zone of adoption |
| --- | --- | --- | --- | --- | --- |
| ICE 15654 A | Desi | 100 | Small | Heat tolerant | Germplasm line |
| JG 14 | Desi | 100-105 | Bold | Heat tolerant | Central India |
| IPC-06-11 | Desi | 100 | Bold | Heat tolerant | Germplasm line |
| MNK-1 | Kabuli | 100-105 | Bold | Heat tolerant | South Zone |
| Pusa-1003 | Kabuli | 110-115 | Small | Heat susceptible | Uttar Pradesh, Bihar, Jharkhand |
| Vijay | Desi | 105-110 | Small | Heat susceptible | Maharashtra, Gujarat |
| JG-63 | Desi | 110-115 | Small | Heat susceptible | Central India |
| JG 16 | Desi | 110 | Bold | Heat susceptible | Central India |

**Table S2.** Flowering time of different chickpea genotypes in the study site in two consecutive years

| Genotype | Days to 50% Flowering | |
| --- | --- | --- |
|  | Year 1 | Year 2 |
| ICE 15654 A | 48.0^d^ | 49.5^c^ |
| JG 14 | 45.0^e^ | 50.5^c^ |
| IPC-06-11 | 47.0^d^ | 51.0^c^ |
| MNK-1 | 47.5^d^ | 50.0^c^ |
| Pusa-1003 | 58.5^a^ | 59.5^abc^ |
| Vijay | 55.0^bc^ | 59.0^bc^ |
| JG-63 | 56.0^b^ | 61.0^a^ |
| JG 16 | 54.0^c^ | 58.0^b^ |

a-e, different lowercase letters indicate significant treatment difference at p < 0.05 according to Duncan’s Multiple Range Test.

**Fig. S1** Minimum and maximum temperature (Tmin and Tmax, ℃) and cumulative rainfall (Cum. Rainfall, mm) during the crop season of 2023-24 (a) and 2024-2025 (b).

**Fig. S2** Minimum and maximum temperatures during flowering (FL) and 15 days after flowering (15DAFL) in chickpea genotypes. The length of the box and whiskers represents variations within the chickpea genotypes (n = 8). *** *p* < 0.001
